# cognitive complaints in schizophrenia: characteristics and relationship with insight

**DOI:** 10.1192/j.eurpsy.2022.1997

**Published:** 2022-09-01

**Authors:** M. Bouhamed, S. Hentati, A. Bouaziz, A. Guermazi, R. Sallemi, J. Masmoudi

**Affiliations:** Hospital university of HEDI CHAKER, Psychiatry A Department, Sfax, Tunisia

**Keywords:** schizophrénia, insight, cognitive complaints

## Abstract

**Introduction:**

Patients with schizophrenia suffer from cognitive difficulties expressed in the form of complaints as well as poor insight. The interaction of these two factors makes the management of these patients more difficult.

**Objectives:**

To assess subjective cognitive complaints in a population of schizophrenics and study its relationship to insight.

**Methods:**

Our study was a cross-sectional, descriptive, and analytical study of 72 stabilized schizophrenics followed up at the outpatient clinic. Subjective cognitive complaints were assessed by the SSTICS, clinical symptoms by the PANSS, and insight by the SAI-E.

**Results:**

The mean age of our population was 46.83± 11.6 years. The patients had a low socio-economic level in 70.1%. They were unemployed in 46.9% , consumed alcohol in 23.6%, and consumed tobacco in 58,6% of the cases. the total score on the PANSS scale was 46. They had an average score of 25 on the total SSTICS score and 20,1 on the SAI-E. Cognitive complaint scores were significantly correlated with improved insight (p=0,00),low socio-economic level (p=0.04),alcoholism (p=0.001) and smoking (p=0.01)

**Conclusions:**

Cognitive complaints in schizophrenia could be influenced by the level of clinical insight and reflect a deep malaise, requiring a more targeted and optimized management

**Disclosure:**

No significant relationships.

